# Finite Element Analysis of the Ballistic Impact on Auxetic Sandwich Composite Human Body Armor

**DOI:** 10.3390/ma15062064

**Published:** 2022-03-11

**Authors:** Imtiaz Alam Shah, Rafiullah Khan, Seyed Saeid Rahimian Koloor, Michal Petrů, Saeed Badshah, Sajjad Ahmad, Muhammad Amjad

**Affiliations:** 1 Department of Mechanical Engineering, International Islamic University, Islamabad 44000, Pakistan; imtiaz.alam@iiu.edu.pk (I.A.S.); rafiullah.khan@iiu.edu.pk (R.K.); saeed.badshah@iiu.edu.pk (S.B.); m.amjad@iiu.edu.pk (M.A.); 2 Institute for Nanomaterials, Advanced Technologies and Innovation (CXI), Technical University of Liberec (TUL), Studentska 2, 461 17 Liberec, Czech Republic; s.s.r.koloor@gmail.com; 3 Technical University of Liberec (TUL), Studentska 2, 461 17 Liberec, Czech Republic; michal.petru@tul.cz

**Keywords:** finite element analysis, auxetic sandwich composite, monolithic armor plates, Johnson–Cook model, ultra-high-molecular-weight polyethylene, silicon carbide

## Abstract

In this study, the ballistic impact behavior of auxetic sandwich composite human body armor was analyzed using finite element analysis. The auxetic core of the armor was composed of discrete re-entrant unit cells. The sandwich armor structure consisted of a front panel of aluminum alloy (Al 7075-T6), UHMWPE (sandwich core), and a back facet of silicon carbide (SiC) bonded together with epoxy resin. Numerical simulations were run on Explicit Dynamics/Autodyne 3-D code. Various projectile velocities with the same boundary conditions were used to predict the auxetic armor response. These results were compared with those of conventional monolithic body armor. The results showed improved indentation resistance with the auxetic armor. Deformation in auxetic armor was observed greater for each of the cases when compared to the monolithic armor, due to higher energy absorption. The elastic energy dissipation results in the lower indentation in an auxetic armor. The armor can be used safely up to 400 m/s; being used at higher velocities significantly reduced the threat level. Conversely, the conventional monolithic modal does not allow the projectile to pass through at a velocity below 300 m/s; however, the back face becomes severely damaged at 200 m/s. At a velocity of 400 m/s, the front facet of auxetic armor was destroyed; however, the back facet was completely safe, while the monolithic panel did not withstand this velocity and was completely damaged. The results are encouraging in terms of resistance offered by the newly adopted auxetic armor compared to conventional monolithic armor.

## 1. Introduction

Novel auxetic structural materials are starting to be used for subject applications, due to them having several properties supporting the desired objective. Auxetic materials are such a class of materials that have a negative Poisson ratio, showing a counter-intuitive behavior, whereby they become thicker perpendicular to an applied force [1,2,3].

Auxetic materials have improved mechanical properties compared with conventional ones, such as shear resistance, indentation resistance, fracture resistance, synclastic behavior, variable permeability, and energy absorption [1,4,5,6,7]. These materials are utilized in various structural applications in aerospace, automotive, biomedical, composite, defense, sensors/actuators, and textile industries [1,2,8,9]. In the aerospace industry, auxetic materials are used in the vanes of the gas turbine in aircraft engines. They are also used in aircraft nose cones and wing panels. In automobiles, they have been widely used in vehicle bumpers and mechanical fasteners [10]. In biomedical applications, they are used in bandages, artificial vessels, dental floss, and surgical implants, having similar properties to bone. In the textile industry, the auxetic fabric is formed by auxetic yarns to result in properties having anti-odor and anti-inflammatory effects [9,11]. In safety devices, auxetic foams are used in helmets, human vests, and knee pads protecting against the ballistic impact in the sports industry, and auxetic re-entrant cells are employed in blast-resistant structures against explosives [11,12,13].

Ballistics may easily be defined as the impact of a comparatively small object/projectile on a heavy body [14,15]. As ballistic threats are increasing with developments in the arms industry, there is a need for novelty in armor to capture their impact energy, with the least effect on the back facet associated with the human’s chest [16,17]. The traditional method employed in armor was the usage of thick steel plates having a high weight and cost, but multi-layered armor was developed with an improved resistance/weight ratio compared to steel, to protect against projectiles with sufficient impact energy [18]. With improvements in the armory industry, hybrid composite structures have been developed for ballistic threats, where bonded material plates need to maintain their individual properties, but cohesively, their response would be far better than individual ones [8,13,19,20].

Auxetic materials are available in different cellular geometries, composed of a combination of unit cells and prepared through various techniques. Commonly available auxetic cellular unit cell geometries include honeycomb, re-entrant auxetic, auxetic-strut, auxetic-honeycomb1 (AH-V1), and auxetic-honeycomb2 (AH-V2) [2,21,22,23].

Imbalzano et al. [24,25,26,27,28] investigated the impact of a sandwich composite with re-entrant cellular auxetic core geometry against impulsive blast loading, both experimentally and using finite element analysis (FEA). They concluded that the armor model reduces the maximum velocity on a back facet by up to 70% and displacement by up to 30% due to densification and plastic deformation of the auxetic cores, compared to monolithic plates of the same dimensions and characterizations. Steven Linforth [29] investigated the response of armor with oval cellular geometry as an auxetic core both experimentally and using FEA and concluded that the armor with a sandwich auxetic core showed a response up to expectations, due to densification and load distribution by the auxetic core.

Novak et al. [30] studied blast and ballistic loading of an auxetic sandwich composite structure with chiral cellular auxetic as its core in between, supported from both ends by alloys of aluminum 7075-T651 and titanium Ti-Gr.37 as cover plates, both experimentally and using FEA (LS-DYNA). The structure was subjected to a fragment-simulating projectile of grade 4340 steel, with different velocities around 300 m/s using a gas gun. They concluded that auxetic sandwich panels have better energy absorption than monolithic plates. Similarly, the experimental and simulation results were in good agreement with each other. Shu Yang et al. [31] performed a comparative study of the ballistic resistance of sandwich panels of aluminum foam and auxetic honeycomb cored sandwich structures. A simulation study was performed on the perforation resistance of auxetic cored sandwich panels targeted by a high-impact projectile, which was compared with aluminum foam cored sandwich panels of similar dimensions. They observed that the panels with an auxetic core were more effective than those with aluminum foam due to material concentration at the impacted area caused by their negative Poisson ratio. After modifying various parameters, such as impact velocity, thicknesses of the faces, core, and core density, they found that the armor with an auxetic core had far better energy absorption than monolithic panels.

The literature shows the impact resistance behavior of composites having auxetic cores of re-entrant and oval unit cell geometries under blast impact, where the lower body structure of armored military vehicles is composed of auxetic cored geometries, in avoiding threats offered by improvised explosive devices. That is why auxetic materials have attracted the attention of researchers keen to explore their resistance to ballistic impact. To employ auxetic materials as body armor, the investigation of ballistic impact behavior is of prime importance. In the current study, the aim was to investigate the behavior of re-entrant auxetic cored sandwich structures for their use in bullet-resistant armor by comparing them with different armor keeping the same thickness and boundary conditions. If the front facet plate is targeted by a projectile with an appropriate velocity, then its impact will directly affect the back facet plate, and ultimately a human’s chest, while in sandwich cored armor, it will behave differently due to the sufficient deformation resistance offered by the struts of the unit cells of the auxetic core.

FEA of newly adopted sandwich panel armor with an auxetic core of re-entrant unit cell was performed on the absorption of impact energy of a projectile using the Johnson–Cook plasticity and failure models. The velocity of the projectile was varied, and the results were compared with the results of simulations of armor with monolithic panels. The following section describes the details of the FEA used in the current study. Section 4 presents the results of the analysis undertaken, followed by the conclusion of the research.

## 2. Materials and Methods

### 2.1. Numerical Model of Armor and Projectile

**Figure 1:**
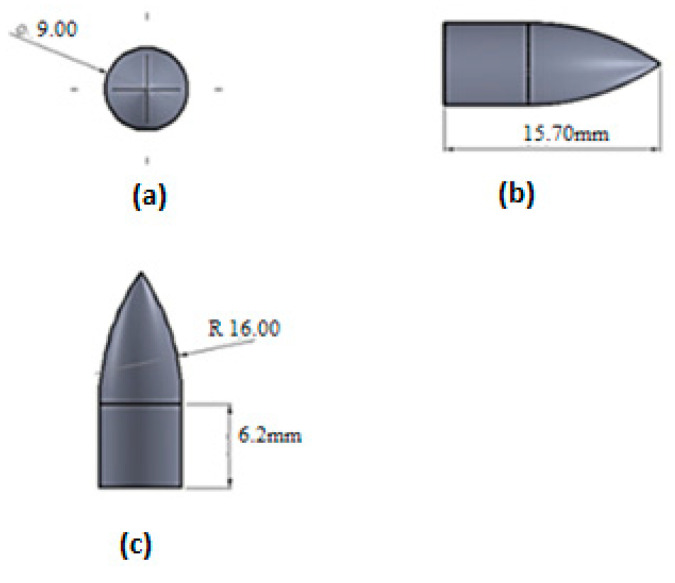
Projectile model with dimensions. (**a**) Front, (**b**) side, and (**c**) top views [19].

In this research, a hybrid structure was composed of an auxetic core sandwiched in between front and back facet plates. The projectile was modeled using the dimensions given in Figure 1. The mass of the projectile used in the analysis was 7.8 × 10^−3^ kg.

**Figure 2:**
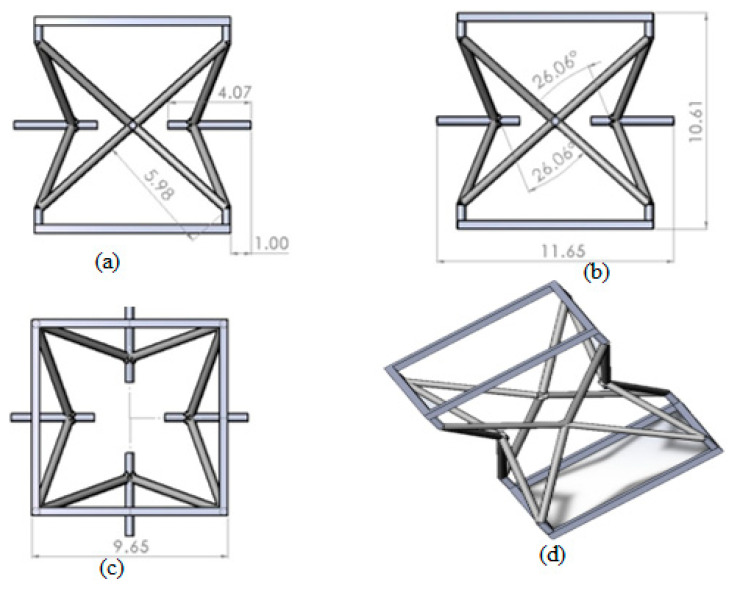
Schematic design of a re-entrant unit cell for an auxetic core with (**a**) front, (**b**) side, (**c**) top view, and (**d**) isometric views.

The auxetic core was built from discrete re-entrant unit cells using 3D modeling software (Solidworks19). The parametric geometry of the unit cell is shown in Figure 2.

**Figure 3:**
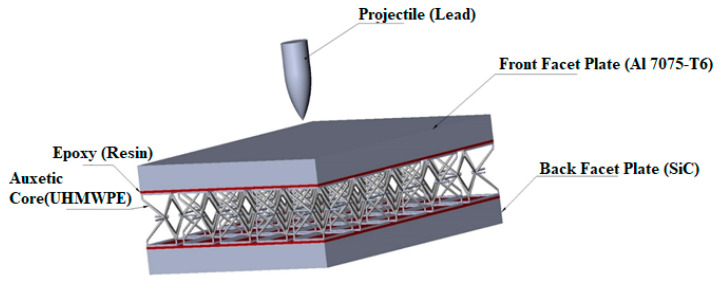
Detailed configuration of the armor structure.

**Figure 4:**
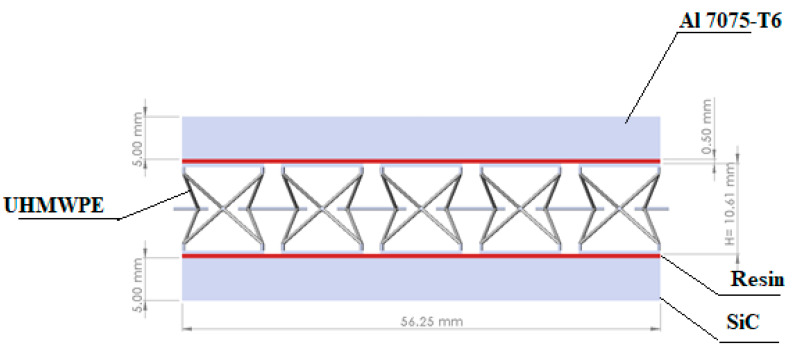
Structure of the armor with dimensions.

The complete structure of the armor in this study was composed of an auxetic core material, ultra-high-molecular-weight polyethylene (UHMWPE), with a front facet panel of an aluminum alloy (Al 7075-T6), and a back facet panel of silicon carbide (SiC). The core was glued with face panels with epoxy resin to avoid sliding and separation. The epoxy resin with 0.5 mm thickness was provided to join the core and face panel. The Shock Equation of state (linear) was used to compute the stresses and strain in the core and resin. The complete structure geometry of the armor is shown in Figure 3 and Figure 4. The mechanical properties of Al 7075-T6, UHMWPE, SiC, and epoxy resin are given in Table 1.

### 2.2. Impulse–Momentum and Kinetic Energy

The relationships between various key parameters, such as mass, velocity, and forces were described by Sir Isaac Newton in his laws for interacting bodies. Similarly, the laws of conservation of energy and momentum are best explained through balancing equations [19].

The impulse–momentum relationship states that the momentum of interacting bodies is equal to impact force times “*t*”. Mathematically:

(1) $$\int_{ti}^{tf}Fdt=m\left(V_{i}-V_{f}\right)$$

while kinetic energy is given by the law of conservation of energy.

(2) $$\frac{1}{2}m\left(V_{i}2-V_{f}2\right)=E_{damping}+E_{elastic}+E_{plastic}+E_{Kinetic}.\;$$

If a projectile is rigid, i.e., non-deformable, then its heat dissipation, acoustic, and other rotational energies can be ignored, after simplification:


(3) $$E_{Kinetic}=\frac{1}{2}m\left(V_{i}2-V_{f}2\right)$$

### 2.3. Johnson–Cook Plasticity Model

Johnson and Cook suggested a semi-experimental model for materials undergoing high strain, strain rates, and temperatures, which in every case the terms “strain hardening, strain rate hardening, and thermal softening” are proposed [32]. After the multiplication of all such terms, flow stress as a function of effective plastic strain “*ε_p_*”, effective plastic strain rate “($\dot{\varepsilon_{p}}$)”, and temperature “*T*” are found. Yield stress is mathematically attained as:

(4) $$\sigma_{y}=\left[A+B\varepsilon_{p}^{n}\right]\left[1+Cln\left(\frac{\dot{\varepsilon_{p}}}{\dot{\varepsilon_{0}}}\right)\right]\left[1-{\left(\frac{T-T_{r}}{T_{m}-T_{r}}\right)}^{m}\right]$$

where “*A*” is the initial yield stress, “*B*” is a strain hardening co-efficient, “*n*” is the strain hardening exponent, $\dot{\varepsilon_{p}}$ is plastic strain rate, $\dot{\varepsilon_{0}}$ is the reference strain rate, and “*C*” is the reference strain rate co-efficient. Here, “*m*” denotes thermal softening. The Johnson–Cook model parameters for Al 7075-T6 are listed in Table 2.

### 2.4. Johnson–Cook Failure Model

The failure of material occurs due to strength degradation, strain energy dissipation, loading, and thermal and mechanical effects [8,37,38,39,40]. Johnson and Cook proposed a failure model using strain rate and temperature, which affect the fracture strain [33]. These parameters/variables are an integral part of their proposed model. The Johnson–Cook failure model has a damage parameter *D*:

(5) $$D=\frac{\varepsilon_{p}}{\varepsilon_{i}^{f}}$$

where $\varepsilon_{p}=\int_{t=0}^{t}{\dot{\varepsilon }}_{p}dT$, with fracture strain given as follows:

(6) $$\varepsilon_{p}=\left(D_{1}+D_{2}exp\left(D_{3}\sigma^{*}\right)\right)\left(1+D_{4}ln\dot{\varepsilon_{p}^{\prime }}\right)\left(1+D_{5}T^{\prime }\right)$$

where σ* is the stress triaxiality, which is σ* = *σ_h_/σ_e_*, *σ_h_* is hydrostatic stress, and *σ_e_* is effective stress, while *D*_1_, *D*_2_, *D*_3_, *D*_4_, and *D*_5_ are the damage parameters of the material under consideration. *D*_1_, *D*_2_, and *D*_3_ are determined from the curve fitting of equivalent plastic strain plotted against the stress triaxiality. Similarly, *D*_4_ and *D*_5_ are determined from the curve fitting of equivalent plastic strain plotted against the strain rate and temperature, respectively [36,41]. The Johnson–Cook failure model parameters for Al 7075-T6 are given in Table 3.

## 3. Pre-Processing of the Model

**Figure 5:**
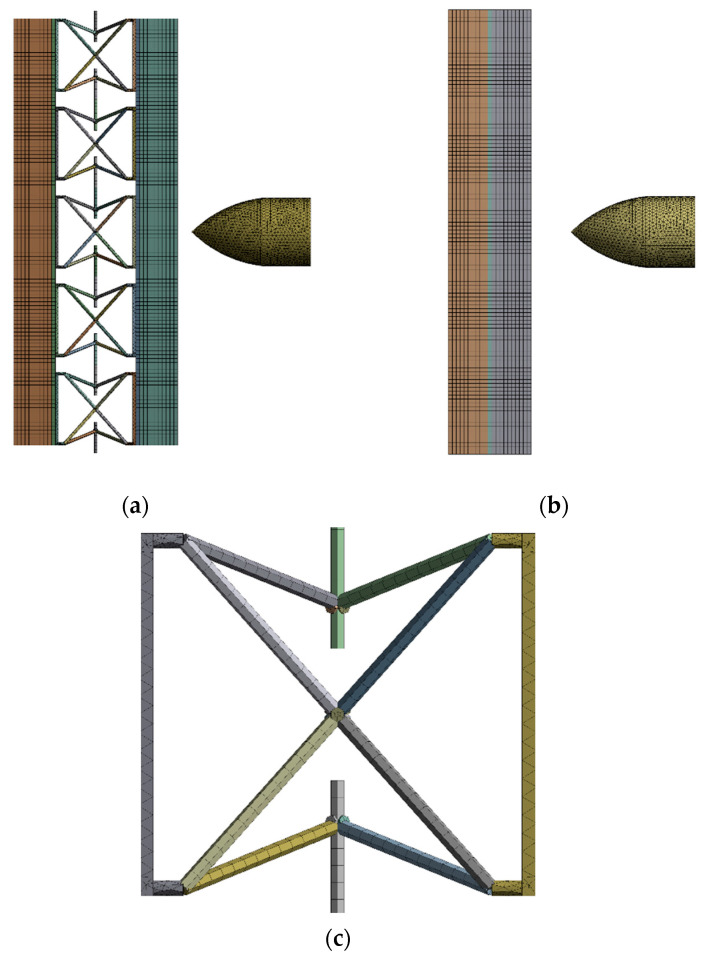
(**a**) Sandwich model with an auxetic core, (**b**) conventional monolithic plate armor (**c**) detailed view of the auxetic core.

The model for the conventional monolithic and auxetic materials was a strike at the center of the plate. The model dimensions are given in Figure 4. The model was converged with a fine tetrahedral and hexahedral mesh with solid elements for auxetic armor models, with 463,835 elements and 418,512 nodes. The mesh sensitivity tests are shown in Table 4 for the striking velocity of 200 m/s. Since there is no significant difference between test 5 and test 6, the number of elements in test 5 was used for the simulation to reduce the computational time. The auxetic core mesh consisted of hexahedral and tetrahedral solid elements. Similarly, for the monolithic material, the mesh was converged with 318,324 elements and 300,284 nodes. The monolithic model with a thickness of 10.5 mm was used in the meshed model. Figure 5 shows both the meshed armor models for the simulation. In Figure 5c, the detailed view of the auxetic core is presented.

To assess energy conservation, directional deformation along with the projectile motion, and stresses withstood by the armor models, several simulations were run. The simulations maintained the geometry and other parameters except for the velocity, which was varied from 100 to 600 m/s.

Numerical simulations of the armor models were run using ANSYS-19R1 software on Explicit Dynamics/Autodyn-3D Lagrangian code, with Johnson–Cook plasticity and failure models. The results of the simulation are given in the next section.

## 4. Results and Discussion

**Figure 6:**
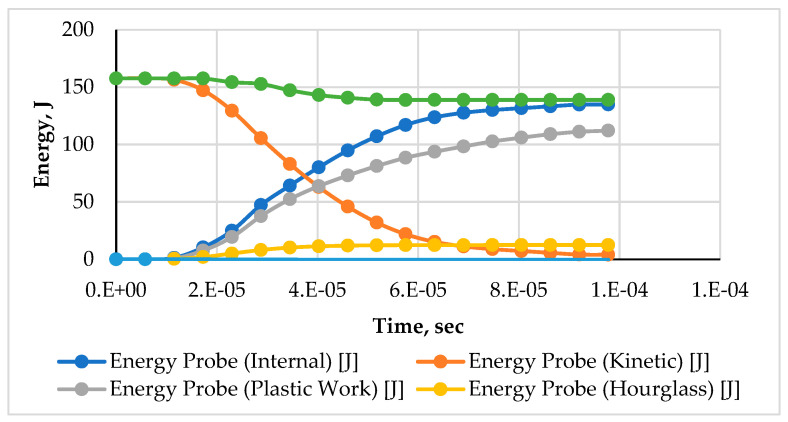
The typical history of energy conservation for impact applications at 200 m/s of an armor with an auxetic core.

**Figure 7:**
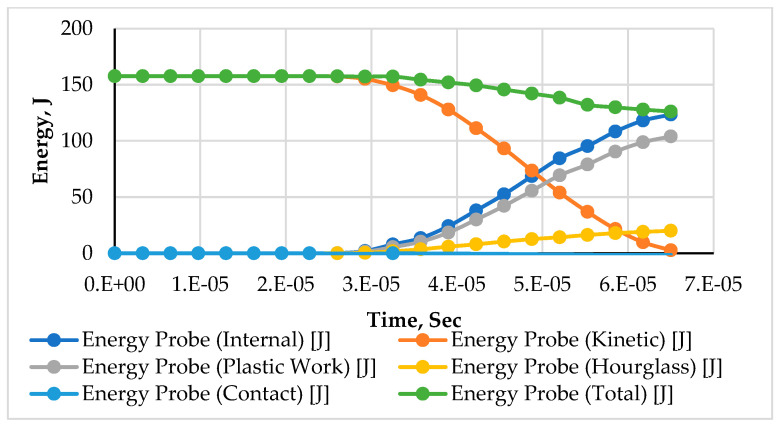
The typical history of energy conservation for impact applications at 200 m/s of an armor with monolithic panels.

The graphs in Figure 6 and Figure 7 show that upon the impact of the projectile with the armor, its kinetic energy drops in a non-linear, exponential trend. The decrease in kinetic energy is transformed into an increase in internal energy. This transformation follows the law of conservation of energy, as the rate of decline in kinetic energy is equal to the increase in internal energy and loss of energy along with the separated elements. The slight decrease in the total energy is due to the detached element. The energy associated with these elements causes an overall reduction in the total energy.

The typical energy transformation in the two models made with the different structural compositions (auxetic and monolithic) in Figure 6 and Figure 7 show significant variations. The drop in kinetic energy observed in the auxetic model started earlier at around 1.15 × 10^−5^ s compared to 2.6 × 10^−5^ s in the monolithic model. This is due to the property of enhanced energy absorption capability offered by the auxetic core. The kinetic energy of the projectile was transformed into internal energy (elastic and plastic work). The difference in the values observed in the internal energy and plastic work of both models shows the distinct behavior of the material.

**Figure 8:**
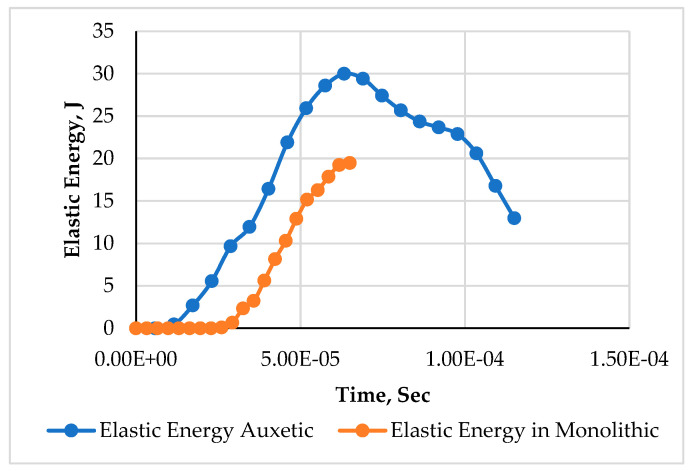
Elastic energy variation for impact applications at 200 m/s of armor with auxetic and monolithic panels.

To study this difference, the elastic energy dissipation during projectile impact was plotted for both models, shown in Figure 8. The elastic energy dissipation was higher the entire time in the phenomenon of impact for the auxetic model, as compared to the monolithic model. Quantitatively, the difference observed at 6.5 × 10^−5^ s was around 33%. After this peak value, the elastic energy in the auxetic model declined gradually as the projectile penetration intensified, causing an increment in plastic work. The higher value of elastic energy dissipation signifies the resistance against penetration, comparatively, resulting in the least damage to the material.

**Figure 9:**
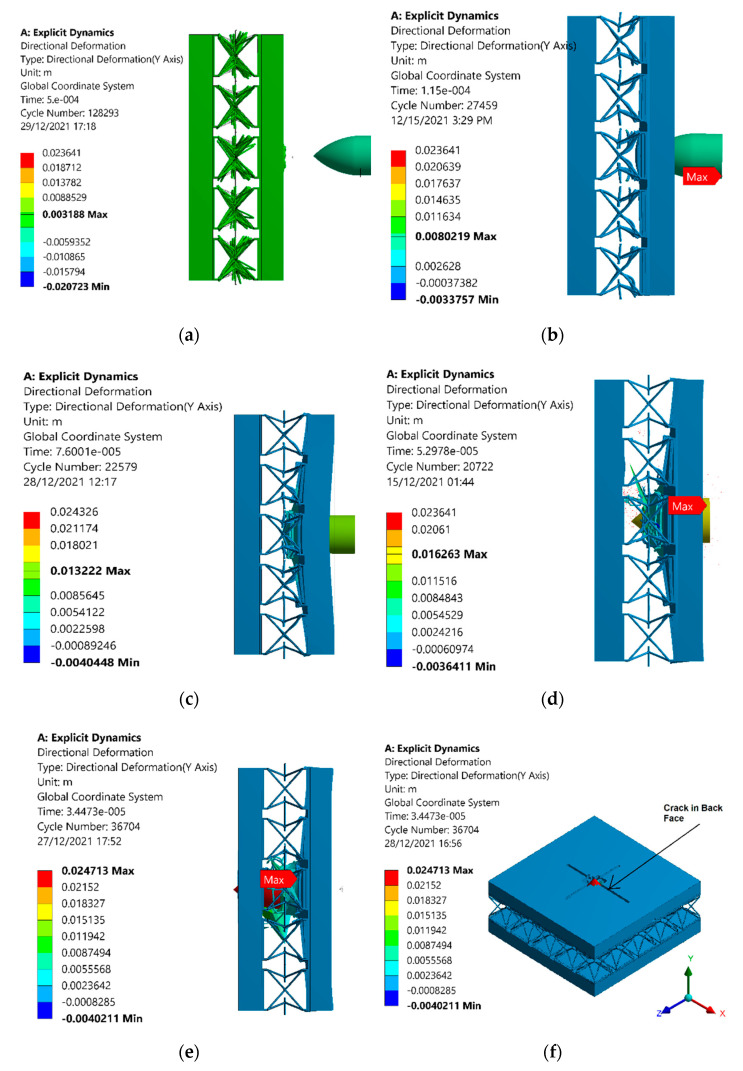
Simulation results of deformation patterns of the sandwich armor with a re-entrant auxetic core under different projectile velocities of (**a**) 100, (**b**) 200, (**c**) 300, (**d**) 400 (**e**) 600 m/s, and (**f**) 600 m/s, isometric view.

It was shown that due to the sufficient densification and indentation resistance offered by the auxetic core, the projectile cannot penetrate the back facet plate, up to a projectile velocity of 400 m/s, as shown in Figure 9. Figure 9 shows the penetration of the projectile and the response behavior of the auxetic core. In Figure 9a, the penetration is least, and energy absorption is mostly elastic shown by the deformation patterns in the auxetic cells. The front face was observed to have been slightly damaged after the impact of the projectile. The auxetic cells could be observed to vibrate frequently as far as the projectile is bounced back. Increasing the velocity of the projectile led to higher kinetic energy, which increased the penetration of the projectile, as shown in Figure 9b. The incremental increase in penetration can be observed in Figure 9c at 300 m/s, Figure 9d at 400 m/s, and Figure 9e at 600 m/s. The rear face of the front plate was observed with significant deformation due to elastic energy dissipation in the unit cells of the auxetic core. The maximum directional deformation value at 100 m/s was noted as 3.188 mm and 8.02 mm at 200 m/s. These values continued to increase as the velocity increased; at 300 m/s, the directional deformation value was 13.22 mm, and at 400 m/s, the value was 16.263 mm. The energy absorption in the auxetic core resulted in comparatively least damage to the material, and the projectile did not reach the back face of the panel up to 400 m/s. At 600 m/s, the kinetic energy sufficiently increased, and hence penetrated the back face of the panel, as shown in Figure 9e,f.

**Figure 10:**
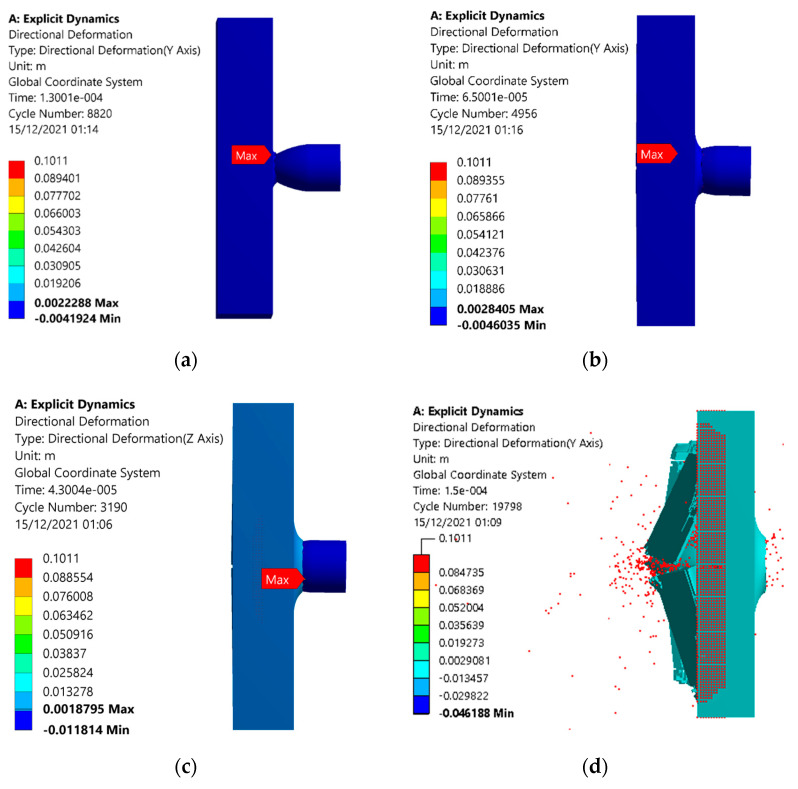
Simulation results of deformation patterns of the conventional monolithic panel armor under different projectile velocities of (**a**) 100, (**b**) 200, (**c**) 300, and (**d**) 400 m/s.

**Figure 11:**
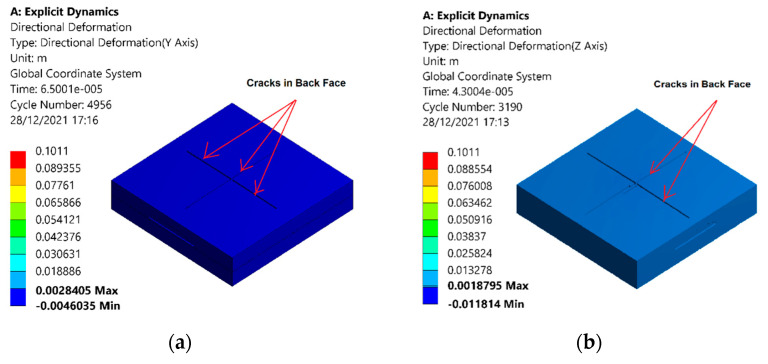
Simulation results of deformation patterns of the conventional monolithic panel armor under different projectile velocities of (**a**) 200, (**b**) 300 m/s with visible cracks in the back face.

In the case of the monolithic plates, the back facet panel was damaged at and above a velocity of 200 m/s, as shown in Figure 10 and Figure 11. The deformation contours of monolithic panel showed an increase in penetration depth with an increase in velocity. At a velocity of 100 m/s, the deformation of 2.22 mm was noted in the direction of projectile motion, as shown in Figure 10a. Furthermore, this increased to 2.845 mm at a projectile velocity of 200 m/s. The value of deformation was reduced to 1.87 m at 300 m/s, as some of the energy was dissipated in cracking the plate, although the projectile was not able to pass through the plate. At 400 m/s, the panel was completely damaged, and the projectile passed through it. Even at a lower velocity of 200 m/s, the plate was cracked, reducing the reliability of the monolithic panel, as shown in Figure 11a,b.

**Figure 12:**
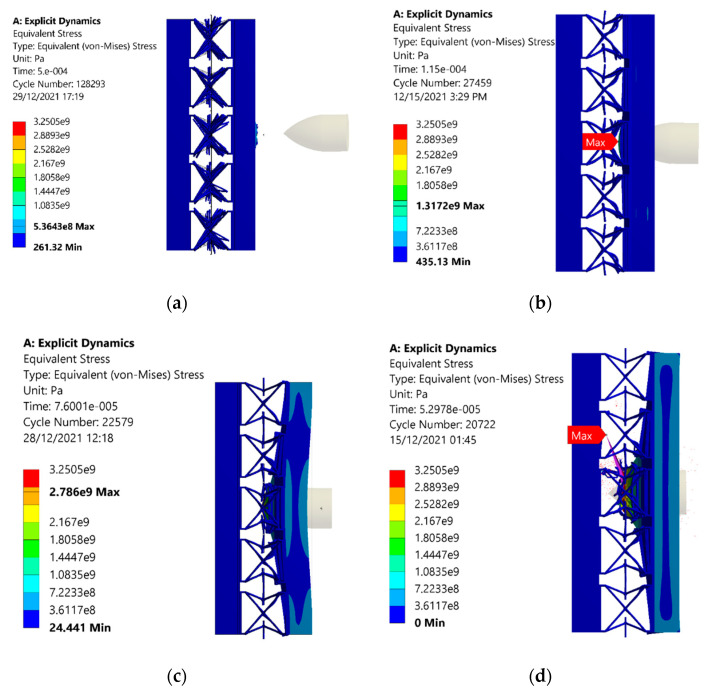

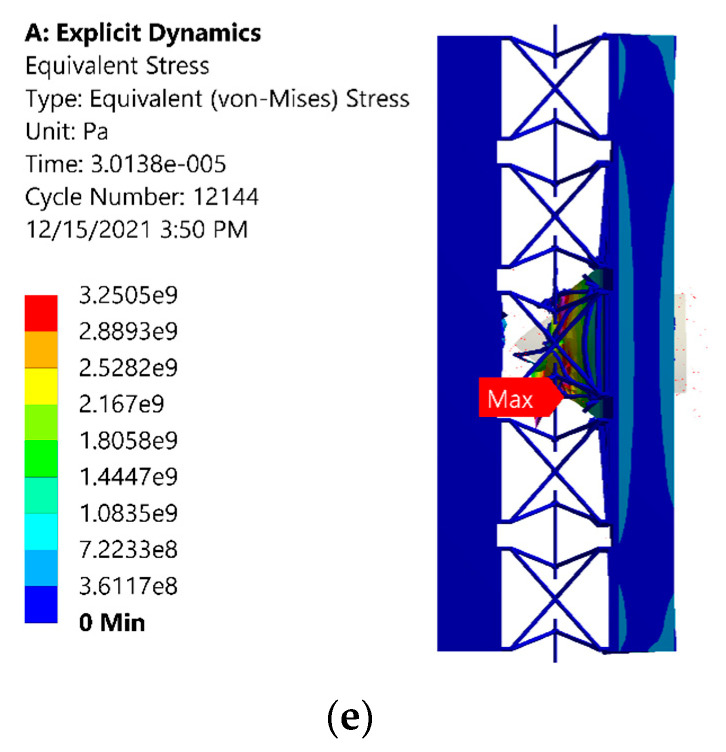
Stresses withstood in the sandwich armor with an auxetic core due to the impact of the projectile under different velocities of (**a**) 100, (**b**) 200, (**c**) 300, (**d**) 400, and (**e**) 600 m/s.

The contours for equivalent stresses of the sandwich structure with an auxetic core are shown in Figure 12. The value of the stresses was minimum for the least velocity of 100 m/s and increased with an increase in the velocity of the projectile. The maximum stress value of 536 MPa was noted in the 100 m/s velocity case, as shown in Figure 12a. When the velocity increased to 200 m/s, the impulse force increased, resulting in the stress reaching a value of 1317 MPa, as shown in Figure 12b. The higher value of the stress resulted in the removal of elements of the material. Further increases in velocity led to higher values of stress, shown in Figure 12c–e.

**Figure 13:**
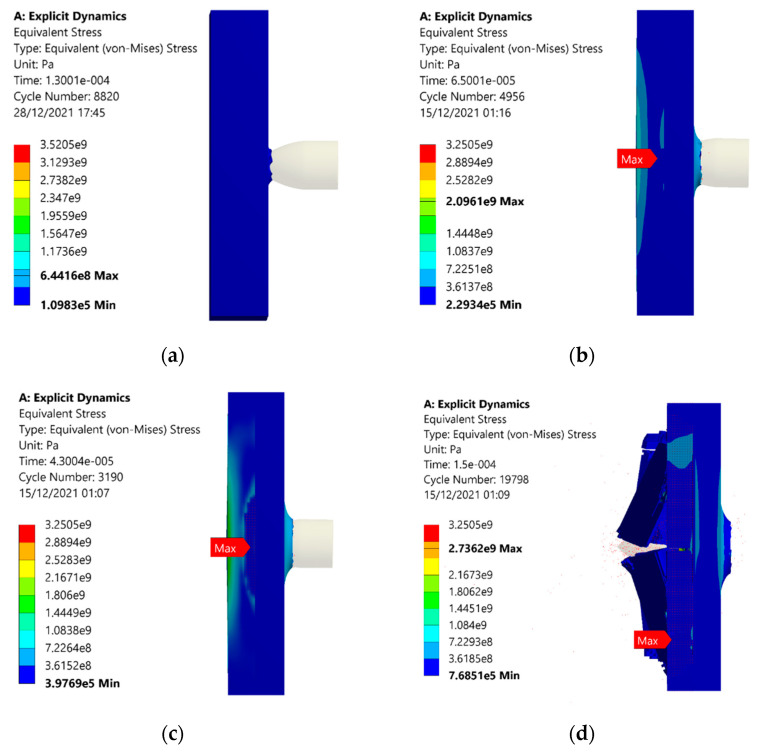
Stresses withstood in the armor with monolithic panels due to the impact of the projectile under different velocities of (**a**) 100, (**b**) 200, (**c**) 300, and (**d**) 400 m/s.

The equivalent stresses in the monolithic panel are plotted, as shown in Figure 13. The stress values in each case are higher than that of sandwich structures with an auxetic core. At 100 m/s, the equivalent stress of 644 MPa is noted, shown in Figure 13a, which is higher than the value of 536 MPa shown in Figure 12a. The reason for the comparatively higher value of stresses is that elastic energy dissipation is less than auxetic material. Similar trends for stresses are observed, such as in auxetic structures. Stress values increased with the projectile velocity of 200 m/s, shown in Figure 13b, and then further increased when the velocity increased. At 400 m/s, the stress reduced as the energy was released, when the material was damaged, as illustrated in Figure 13d.

## 5. Conclusions

In this study, the ballistic impact behavior of auxetic sandwich composite armor was investigated. The auxetic core was made up of discrete re-entrant unit cells. The impact of a projectile on the auxetic sandwich composite armor at different velocities was simulated in ANSYS 19R1. The results were compared to monolithic armor with the same velocity and boundary conditions. The following conclusions were drawn from the research:

1. The analysis shows a higher energy transformation of the kinetic energy of a projectile in elastic energy occurs in auxetic structure compared with the monolithic panel due to the elastic deformation of unit cells in the auxetic core. The higher absorption capability of the auxetic structure makes it dominant over the monolithic panel.
2. Deformation induced in the auxetic model is higher compared with the monolithic panel; however, this deformation does not affect the backplate. The elastic energy dissipation is higher in the auxetic structure. The auxetic structure is safe up to 400 m/s and can be used at a higher velocity, significantly reducing the threat level. At 600 m/s, the back face is damaged.
3. Due to significant deformation and indentation resistance offered by the struts of the discrete unit cells of the auxetic core, the transformation of stresses is minimized and does not transmit into the back facet plate for projectiles fired at a velocity of 400 m/s.
4. The monolithic panel gets damaged at a lower velocity of 200 m/s and is completely damaged at 400 m/s, showing lower resistance to indentation as compared to auxetic structures.

## Figures and Tables

**Table 1:** Material properties of Al 7075-T6, UHMWPE, SiC, and epoxy resin.

Sr. No.	Material	Property	Value	Unit
1	Aluminum alloy (Al 7075-T6) [32]	Density	2804	kg/m^3^
		Specific Heat, C	848	J/kg °C
		Shear Modulus	2.67 × 10^10^	Pa
2	UHMWPE [33]	Density	915	kg/m^3^
		Shear Modulus	1.7 × 10^8^	Pa
3	SiC [34]	Density	3215	kg/m^3^
		Specific Heat, C	510	J/kg °C
		Shear Modulus	1.935 × 10^11^	Pa
		Bulk Modulus	2.2 × 10^11^	Pa
4	Epoxy resin [35]	Density	1186	kg/m^3^
		Shear Modulus	2.1 × 10^9^	Pa

**Table 2:** Johnson–Cook strength and failure model parameters for Al 7075-T6 [32,36].

Johnson–Cook Strength Model			
Sr. No.	Property	Value	Unit
1	Strain Rate Correlation	First-Order	
2	Initial Yield Stress	5.46 × 10^8^	Pa
3	Hardening Constant	6.78 × 10^8^	Pa
4	Hardening Exponent	0.71	
5	Strain Rate Constant	0.024	
6	Thermal Softening Exponent	1.56	
7	Melting Temperature	893	K
8	Reference Strain Rate (/sec)	0.0005	

**Table 3:** Johnson–Cook failure model parameters for Al 7075-T6 [36,41].

Sr. No.	Property	Value
1	Damage Constant *D*_1_	−0.068
2	Damage Constant *D*_2_	0.451
3	Damage Constant *D*_3_	−0.952
4	Damage Constant *D*_4_	0.036
5	Damage Constant *D*_5_	0.697
6	Melting Temperature	893 K
7	Reference Strain Rate (/sec)	1

**Table 4:** Mesh sensitivity tests.

Test No	No of Elements	Equivalent Stress, Pa
1	249,342	6.12 × 10^8^
2	316,473	1.01 × 10^9^
3	358,952	1.15 × 10^9^
4	404,591	1.27 × 10^9^
5	463,835	1.32 × 10^9^
6	524,795	1.32 × 10^9^

## Data Availability

The data presented in this study are available on request from the corresponding author.

## List of Abbreviations

FEA	Finite Element Analysis
UHMWPE	Ultra-High-Molecular-Weight Polyethylene
SiC	Silicon Carbide
AH	Auxetic Honeycomb
NPR	Negative Poisson Ratio
JC	Johnson–Cook

## Symbols

F	Impact Force
t	Time
V_i_	Initial velocity
V_f_	Final velocity
E_damping_	Damping Energy
E_elastic_	Elastic Energy
E_plastic_	Plastic Energy
E_kinetic_	Kinetic Energy
σ_y_	Yield Stress
A	Initial Yield Stress
B	Strain Hardening Coefficient
${\dot{\varepsilon }}_{p}$	Plastic Strain Rate
${\dot{\varepsilon }}_{0}$	Reference Strain Rate
C	Reference Strain Rate Coefficient
m	Thermal Softening
D	Damage Parameter
T	Temperature
σ^*^	Stress Triaxiality
